# Conflict resolution strategies and their association with perceived stress among German medical students: a cross-sectional study

**DOI:** 10.3389/fmed.2026.1846823

**Published:** 2026-06-18

**Authors:** Rebecca Reichel, Teresa Festl-Wietek, Tobias Albrecht, Andreas J. Fallgatter, Anne Herrmann-Werner

**Affiliations:** 1 Department of Psychiatry and Psychotherapy, University Hospital Tuebingen, University of Tuebingen, Tuebingen, Germany; 2 Tuebingen Institute for Medical Education (TIME), University of Tuebingen, Tuebingen, Germany; 3 German Center for Mental Health (DZPG), Tuebingen, Germany

**Keywords:** communication, conflict, psychological, education, medical, undergraduate, interdisciplinary communication, stress

## Abstract

**Background:**

Effective conflict management is essential in medical teams to maintain collaboration and ensure high-quality patient care. While teamwork, self-reflection, and conflict management are recognized as important competencies in medical education, little is known about the conflict resolution strategies used by medical students or how these strategies are associated with perceived stress, particularly within the context of German medical education. Conflicts in medical training may increase stress and impair teamwork, highlighting the need for targeted educational interventions.

**Aim:**

This study examined conflict resolution strategies among medical students and their association with perceived stress.

**Methods:**

A cross-sectional survey was conducted with 219 ninth-semester medical students at the Medical Faculty of Tübingen, Germany. The German versions of the Conflict Inventory and the Perceived Stress Questionnaire (PSQ-20) were used to measure conflict management styles and stress levels. Data analysis included generalized ordinal regression analysis.

**Results:**

Students predominantly preferred integrative conflict resolution strategies such as collaboration (*mean score = 4.2, SD = 0.5*) and compromise (*mean score = 3.9, SD = 0.6*), whereas competing was the least preferred strategy (*mean score = 2.7, SD = 0.8*). In the regression analysis, team conflicts were associated with higher levels of perceived stress (*B = 3.670, ß = 0.23, p < 0.001*) during undergraduate medical training, and individual conflict resolution tendencies influenced perceived stress levels, with a greater tendency to compromise contributing to lower perceived stress (*B = −5.612, ß = −0.180, p = 0.025*).

**Conclusion:**

Conflict resolution tendencies are associated with perceived stress among medical students. Integrating reflective conflict management training into medical curricula may support teamwork competencies and stress reduction. Further research should examine the long-term impact of such interventions.

## 1 Introduction

### 1.1 The significance of conflicts in medical teams

Medical teams operate in a complex working environment characterized by high workloads and the need for interprofessional and interdisciplinary collaboration. Conflicts constitute an essential element of team collaboration and have the potential to contribute to team growth (1). Effective conflict management can enhance team dynamics, creativity, and the overall effectiveness of collaboration (2). Conflicts arise when the needs, expectations, goals, or values of team members do not align, leading to disrupted communication within the team (2). Particularly in medical teams, numerous interactions between different professions occur, resulting in the large majority of healthcare workers reporting interpersonal workplace conflicts (2). In instances of frequent and persistently unresolved conflicts, there is a risk of increased stress on team members and, ultimately, a deterioration in patient care (3, 4). This burden can, in turn, lead to burnout and higher rates of resignation among healthcare providers, thereby inflicting additional economic damage (5). This is particularly evident in the German healthcare system, where teams have been described as physician-centered and shaped by formal and informal hierarchies, with studies from German hospitals and interprofessional care settings showing that hierarchical role structures influence communication, teamwork, and perceptions of patient safety (6, 7).

The conceptual causes of conflicts can be divided into three levels: (1) organizational level, (2) interpersonal level, and (3) individual level (8). At the individual level, in addition to perceptual differences, personality traits, personal convictions, and individual conflict management and communication styles—with corresponding tendencies to avoid, normalize, or tolerate conflicts—are relevant (8, 9).

### 1.2 Conflict management interventions

Conflict resolution training within medical teams has been shown to improve perceived confidence in dealing with conflicts, enhance work quality, and reduce the number of legal claims arising from malpractice (10, 11). Interventions for medical students have resulted in a more positive attitude and increased self-confidence regarding conflict management (12, 13).

However, Almost et al. (3) and Mohseni et al. (14) have noted in their reviews that there remains insufficient research regarding interventions and their potential to modify the underlying individual causes of conflict, thereby reducing associated stress.

### 1.3 Conflict management in medical education

The importance of effective conflict management for medical teams is reflected in the EPA (Entrustable Professional Activity) catalog of the American Association of Medical Colleges, in which the “Core EPA,” interprofessional teamwork, is described as working together in a trusting and respectful manner. The ability to resolve conflicts is identified as a prerequisite for interprofessional teamwork (15, 16). The need for guided self-reflection as a central aspect of successful conflict management is emphasized by the Association for Medical Education in Europe, which regards it as the foundation for independent, lifelong learning and the development of professional expertise (17). In Germany, these competencies are anchored in the National Competence-Based Catalogue of Learning Objectives for Medicine (NKLM), which serves as a framework for competency-based medical education. The NKLM describes physicians as members of interprofessional teams and explicitly includes learning objectives related to constructive teamwork, reflection on one’s own and others’ behavior, respectful interaction with team members, and the recognition, analysis, and constructive resolution of interprofessional conflicts (18). Self-reflection is frequently realized in the form of written reflection. Lim et al. (19), in their review, concluded that such reflection promotes learning; facilitates ongoing medical education; conveys moral, ethical, professional, and social standards and expectations; improves patient care; and supports the development of professional identity. In the field of organizational psychology, long-established assessment tools—which can reveal enduring personal tendencies—are used for self-reflection on conflict resolution strategies (20, 21).

## 2 Theory of conflict resolution strategies

**Figure 1:**
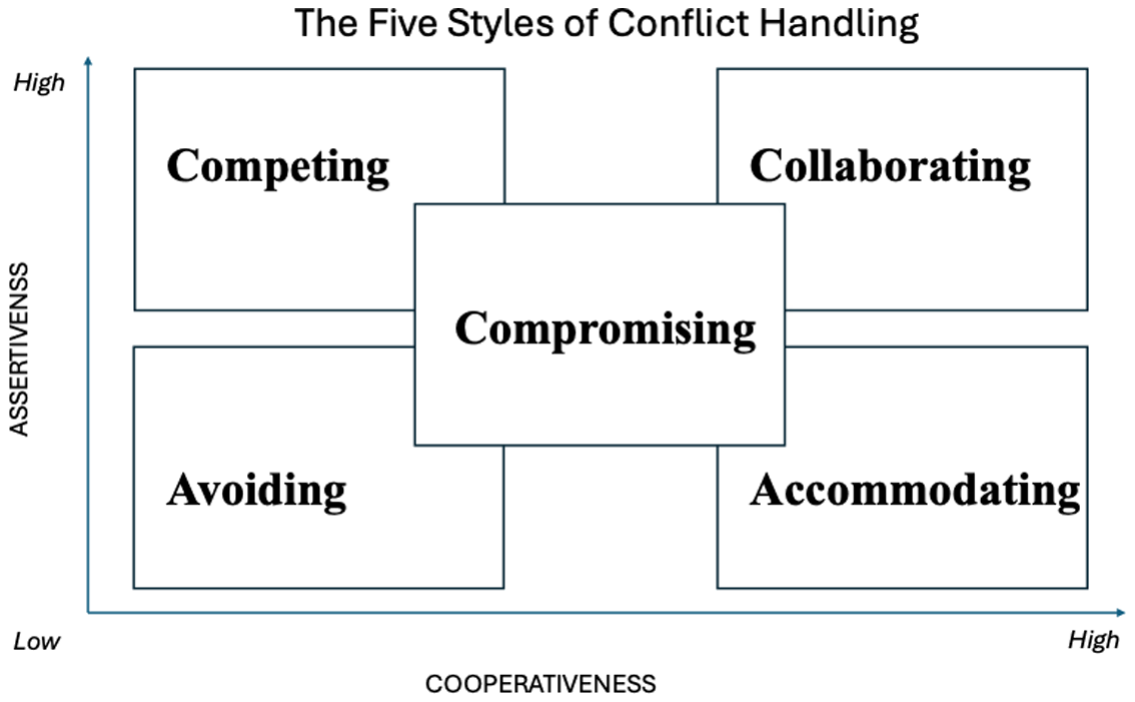
Theory of conflict resolution strategies according to Rahim and Thomas. The figure illustrates the extent of assertiveness versus the interest in collaboration exhibited by the individual conflict resolution strategies, adapted from Thomas and Kilmann (25).

The basis for these assessment tools is the model of modes of conflict resolution (22), which was developed with a focus on the study of conflicts in organizations (23–25). As illustrated in Figure 1, both the Thomas and Rahim models contrast assertiveness with the desire to collaborate (24, 25). All identified conflict resolution strategies are mapped onto these two axes. In total, five fundamental individual approaches to dealing with interpersonal conflicts can be distinguished.

Competing is characterized by the insistence on one’s own concerns, with one’s viewpoint often being maintained uncompromisingly, frequently at the expense of others. The approach of collaborating focuses on addressing the concerns of both parties. Through intensive collaboration and thorough engagement with the issues, alternative solutions that are satisfactory to all involved are pursued. Compromising seeks a balance between competing and accommodating, whereby both sides achieve partial satisfaction without reaching a definitive solution. Avoidance is neither assertive nor cooperative. Conflicts are ignored, and none of the parties involved see their concerns addressed. Accommodating involves a high willingness to cooperate coupled with low assertiveness. The concerns of the other party are fully met, which can range from generosity to sacrificing one’s own interests. Of central importance to the theory is the individual’s flexibility to apply the appropriate conflict style according to the situation. For personal reflection and the improvement of conflict management skills, individual tendencies should be compared with a suitable reference group (25).

The characterization of different conflict resolution strategies has been supported by external assessments in which participants reflected on simulated conflict solutions: The integrative or collaborative conflict style is perceived as the most effective and appropriate. The competing or dominant style is often viewed as inappropriate when used by others, although it is perceived as more effective when used by oneself. The accommodating style is generally assessed as neutral but less effective. The avoiding style is widely considered ineffective and inappropriate. The compromising style is also seen as neutral, although some participants rated their counterparts as more effective and appropriate when they were willing to compromise (26).

Key causes of conflict in medical teams include one’s role in the workplace and the quality of the working environment; personality structure, emotional intelligence, and insufficient support and communication (3, 27). Additionally, studies have shown indications that the individual conflict profile can predict the workplace performance of junior doctors (28) and the perceived work-related stress among nurses (30). According to Friedman et al. (22), conflict styles have a lasting impact on the social environment of employees through the level of ongoing conflict. Specifically, integrative conflict styles have been associated with reduced stress, whereas dominant or avoidant conflict behaviors have been associated with increased workplace stress (22).

In summary, the assessment and self-reflection of conflict resolution tendencies have the potential to enhance the understanding of medical students’ behavior within teams, helping to ensure the quality of work and to contribute to reducing medical students’ perceived stress levels (3).

## 3 Aim of the study

The aim of this study was to gain a deeper understanding of conflict management tendencies among medical students. Specifically, the study addressed the following research questions:

Which conflict resolution strategies do medical students prefer? Do their tendencies influence their perceived levels of stress?

To date, it remains unclear whether the frequent use of particular conflict resolution strategies is associated with higher levels of stress among medical students. The primary hypothesis is that perceived stress levels among participants are influenced by their individual conflict management tendencies.

## 4 Methods

### 4.1 Study design and participants

To examine tendencies in conflict management among medical students and their perceived stress levels, a cross-sectional survey design was chosen. The survey was programmed using SoSci-Survey and included demographic data along with participants’ interest in conflict management, their perception of effective conflict handling as a prerequisite for efficient teamwork, and the extent of subjectively experienced stress due to past team conflict—three Likert-scale items graded from strongly agree to strongly disagree. The three items were developed with the support of experts in the fields of medical education and psychology.

### 4.2 Measurements

Standardized assessments specifically designed for this purpose were used to measure conflict management tendencies and stress levels. These included the German-adapted version of Rahim’s Organizational Conflict Inventory (ROCI-II) and the German version of the Perceived Stress Questionnaire in its short form (PSQ-20) (20, 30). Both assessments were validated in German (20, 30). Moreover, the measurement of conflict management tendencies was shown to be robust against social desirability bias and systematic response distortion (31, 32).

The overall survey design was developed in consultation with experts in the fields of medical education and psychology. To measure general stress perception, the short version of the Perceived Stress Questionnaire (PSQ-20) with the subscales Worries, Tension, Demands, and Joy, which has been widely used in German-speaking countries and shows good reliability with Cronbach’s alpha ranging from 0.80 to 0.86, was used. In this study, Cronbach’s alpha was 0.830. Each subscale consists of five Likert-scale items, in which participants indicate how often (almost never, sometimes, often, or mostly) a given statement applied to their life in the past 4 weeks. The PSQ is considered a valid instrument for recording subjective perceived stress within a transactional model, in which stress reflects the appraisal of demands relative to resources (33).

The German version of the Rahim Organizational Conflict Inventory (ROCI-II-D) comprises 28 self-descriptive items, each rated on a 5-point Likert scale (strongly agree to strongly disagree). The internal consistency reliability coefficient for the ROCI-II ranges between 0.72 and 0.76. The ROCI-II-D provides initial evidence of factorial, convergent, and criterion-related validity. The conflict style structure postulated by Rahim was largely replicated, and the associations with interpersonal problems, individual values, and relationship criteria were predominantly consistent with theoretical expectations. However, the validation should be regarded as preliminary, as the samples were not representative of the general population. Participants were asked to indicate the extent to which the described conflict-handling behavior was typical for them (20). The assessment focused on conflict behavior when interacting with peers. Each item was assigned to a corresponding conflict style based on the described behavior. After completing the assessments, students received feedback on their average conflict management tendencies. Based on their results, they were also provided with personalized self-reflection prompts regarding their conflict behavior.

### 4.3 Data collection

This study used a convenience sample of ninth-semester medical students at the University of Tuebingen. Participants were asked to complete the questionnaire anonymously as part of their curricular coursework in preparation for a psychiatry seminar. Students from the semester cohorts between summer 2022 and winter 2023 were invited to participate in the study to provide their voluntary consent for data analysis by completing the questionnaire. Access to the questionnaire was provided via an external link on the University of Tuebingen’s e-learning platform, allowing anonymous participation before the seminar in a private setting while restricting access to the target group of ninth-semester medical students in Tuebingen. Only completed questionnaires were included in the analysis; no other inclusion or exclusion criteria were applied.

### 4.4 Data analysis

In this model, the conflict management tendencies were treated as independent variables, whereas perceived stress functioned as the dependent outcome variable. The analysis focused on the following subdomains of perceived stress: worry, tension, demands, and joy. In addition, as a secondary hypothesis, the influence of previous experiences with team-related conflicts on students’ perceived stress levels was examined. The survey data were analyzed using IBM SPSS Statistics, version 28. The significance level was set at *α* = 0.05, and the model residuals were normally distributed. Only completed questionnaires were included in the analysis. First, a sociodemographic analysis of the study cohort was conducted based on age and gender. We also examined participants’ interest in conflict management, their perception of effective conflict handling as a prerequisite for efficient teamwork, and the extent of subjective stress experienced due to past team conflict (each measured on a 5-point Likert scale).

The core analysis focused on the descriptive examination of existing conflict management tendencies and their impact on perceived stress within the study cohort. A linear regression analysis was conducted, including ROCI assessment scores, demographic variables (gender and age), and participants’ self-reported stress levels due to previous team conflict. The primary objective was to examine the direction of the influence of conflict management tendencies on participants’ perceived stress levels. The effect of each independent variable was evaluated using the respective regression coefficients. Additionally, regression analyses were conducted for the PSQ subscales: Worries and Joy.

## 5 Results

### 5.1 Demographics

A total of 244 completed questionnaires were collected, of which 219 were included in the analysis. Questionnaires that were not fully completed (i.e., not answered up to the last page) did not provide sufficient data on conflict management tendencies and were therefore excluded. Additionally, responses from participants who did not consent to data evaluation (*n* = 35) were not analyzed. The cohort consisted of 72% (*n* = 157) female participants and 27% (*n* = 59) male participants, with 1% (*n* = 3) choosing not to disclose their gender. The respondents had an average age of 26 years (SD = 3.6), ranging from 21 to 45 years. All participants were in their ninth semester of medical studies at the Eberhard Karls University of Tübingen.

The majority of participants (68%) reported an interest in conflict management (mean score = 3.8, SD = 0.8). Nearly all respondents (96%) agreed that effective conflict handling is a prerequisite for efficient teamwork (mean score = 4.7, SD = 0.6). Meanwhile, 20% of participants indicated that they had experienced subjective stress as a result of past team conflict (mean score = 2.4, SD = 1.2).

### 5.2 Conflict management tendencies

**Figure 2:**
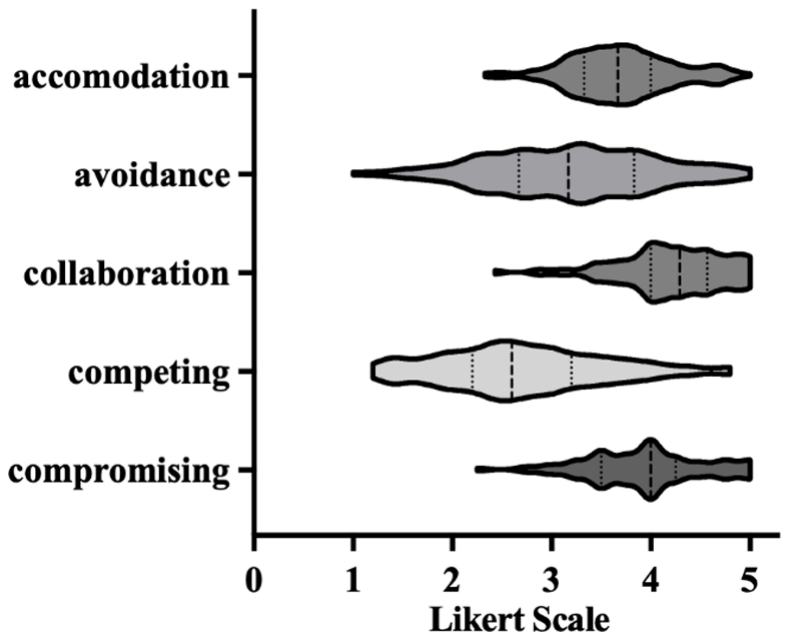
Distribution of conflict management tendencies among German medical students.

An assessment of preferred conflict resolution strategies revealed that the surveyed medical students exhibited tendencies that partially deviated from those of the reference group. The most frequently chosen strategy was *collaboration* (*mean score = 4.2, SD = 0.5*), followed by *compromise* (*mean score = 3.9, SD = 0.6*). Participants were less likely to choose *accommodation* (*mean score = 3.7, SD = 0.5*) or *avoidance* (*mean score = 3.2, SD = 0.8*). The least preferred strategy for conflict resolution was *competing* (*mean score = 2.7, SD = 0.8*). Examining the subgroups by gender, male students showed a slight preference for *competing* (*mean score = 2.9, SD = 0.8*) over *avoidance* (*mean score = 2.9, SD = 0.8*), while female students had a clearer preference for *avoidance* (*mean score = 3.3, SD = 0.8*) over *competing* (*mean score = 2.6, SD = 0.8*) (see Figure 2; Table 1).

**Table 1:** Descriptive statistics of conflict styles by gender.

Gender	*n*	Avoiding *M (SD)*	Collaborating *M (SD)*	Accommodating *M (SD)*	Competing *M (SD)*	Compromising *M (SD)*
Female	15 7	3.3 (0.8)	4.2 (0.5)	3.7 (0.5)	2.6 (0.8)	3.9 (0.6)
Male	59	2.9 (0.8)	4.2 (0.5)	3.6 (0.5)	3.0 (0.8)	3.9 (0.5)

m = mean; SD = standard deviation. Higher scores indicate greater use of the respective conflict style.

### 5.3 Influence of conflict resolution strategies on perceived stress

A multiple linear regression analysis was conducted to examine the influence of demographic factors, previous team conflict, and conflict styles on participants’ perceived stress levels. The analysis of variance (ANOVA) results indicated that the model was statistically significant (*F* (8, 201) = 4.030, *p* < 0.001), suggesting that the independent variables collectively had a significant impact on the dependent variable. The adjusted R^2^ was 0.104, indicating a moderate effect size. As shown in Table 2, to clarify the specific contributions of each variable, the regression coefficient B and the standardized regression coefficient ß were calculated.

**Table 2:** Regression coefficients predicting perceived total stress.

Predictor	*B*	*SE*	*β*	*p*	95% CI for *B*
Age	0.66	0.34	0.13	0.054	[−00.01, 1.34]
Gender	−5.65	2.80	*−*0.14	0.045	[−11.17, *−*0.14]
Stress due to previous team conflict	3.67	1.08	0.23	<0.001	[01.54, 5.80]
Collaboration	1.29	2.88	0.04	0.654	[−04.39, 6.98]
Accommodation	0.39	2.76	0.01	0.887	[−05.05, 5.83]
Avoiding	2.96	1.73	0.14	0.089	[−00.45, 6.37]
Competing	1.06	1.52	0.05	0.486	[−01.94, 4.07]
Compromising	−5.61	2.49	*−*0.18	0.025	[−10.53, *−*0.70]

*B* = unstandardized regression coefficient; *SE* = standard error; *β* = standardized regression coefficient; CI = confidence interval. Dependent variable: PSQ total score.

As demographic influences on perceived stress, the participants’ age and gender were examined. With increasing age, there was a tendency for perceived stress to increase, but the effect fell just below statistical significance (B = 0.663, ß = 0.133, *p* = 0.054). In terms of gender, the analysis showed that female participants experienced significantly higher stress levels (B = −5.652, ß = −0.140, *p* = 0.045). Regarding the impact of previous conflicts, the analysis demonstrated that the burden caused by past team conflicts had the strongest effect of all examined factors (B = 3.670, ß = 0.23, *p* < 0.001). Regarding individual tendencies in conflict resolution strategies, a greater tendency to compromise was associated with significantly lower perceived stress (B = −5.612, ß = −0.180, *p* = 0.025).

In the next step, a regression analysis was conducted for the Worries and Joy subscales, as shown in Tables 3, 4. In summary, conflict resolution tendencies had an influence on the level of worry but had a smaller impact on participants’ sense of joy. More specifically, the stress caused by past team conflict had a reinforcing effect on the level of worry experienced by participants (B = 4.764, ß = 0.252, *p* < 0.001) and a diminishing effect on their sense of joy (B = -4.370, ß = −0.241, *p* < 0.001).

**Table 3:** Regression coefficients predicting perceived worry.

Predictor	B	SE	β	*p*	95% CI for B
Age	0.53	0.39	0.09	0.179	[−00.24, 1.30]
Gender	−5.99	3.20	−0.13	0.062	[−12.29, 0.32]
Stress due to previous team conflict	4.76	1.24	0.25	< 0.001	[02.31, 7.21]
Collaboration	2.73	3.31	0.07	0.410	[−03.79, 9.25]
Accommodation	0.92	3.17	0.02	0.772	[−05.32, 7.16]
Avoiding	5.31	1.98	0.21	0.008	[01.41, 9.21]
Competing	1.03	1.75	0.04	0.556	[−02.41, 4.48]
Compromising	−8.81	2.86	−0.24	0.002	[−14.44,-3.17]

B = unstandardized regression coefficient; SE = standard error; β = standardized regression coefficient; CI = confidence interval. Dependent variable: PSQ Worry.

**Table 4:** Regression coefficients predicting perceived joy.

Predictor	B	SE	β	*p*	95% CI for B
Age	*−*0.31	0.38	*−*0.05	0.413	[−1.06, 0.41]
Gender	5.87	3.12	0.13	0.061	[−0.27, 0.06]
Stress due to previously experienced team conflict	*−*4.37	1.20	*−*0.24	<0.001	[−6.74, *−*0.00]
Collaboration	5.96	3.20	0.15	0.064	[−0.36, 0.06]
Accommodation	3.44	3.09	0.09	0.267	[−2.65, 0.27]
Avoiding	*−*2.78	1.93	*−*0.11	0.150	[−6.58, 0.15]
Competing	1.06	1.70	0.04	0.535	[−2.29, 0.54]
Compromising	4.28	2.75	0.12	0.122	[−1.15, 0.12]

B = unstandardized regression coefficient; SE = standard error; β = standardized regression coefficient; CI = confidence interval. Dependent variable: PSQ Joy.

A higher tendency to *compromise* had a significantly reducing effect on the level of worry (*B = −8.810, ß = −0.238, p = 0.002*), while higher *avoidance* tendencies were associated with increased worry (*B = −4.370, ß = −0.241, p < 0.001*).

Regarding the influence on the sense of joy, an opposite trend was observed for compromising (B = 4.275, ß = 0.119, *p* = 0.112) and avoidance behaviors (B = −2.779, ß = −0.112, *p* = 0.150), although these effects were not statistically significant. The strongest positive effect on the sense of joy was observed for the tendency to collaborate, although this effect also narrowly missed statistical significance (B = 5.956, ß = 0.149, *p* = 0.064).

## 6 Discussion

This study revealed that medical students predominantly preferred integrative conflict resolution strategies, particularly compromising and accommodating, while competitive strategies were used less frequently. In addition, specific conflict resolution styles were associated with higher perceived stress.

### 6.1 Preferred conflict styles

One primary objective was to determine which conflict resolution strategies were preferred by medical students. Overall, the survey revealed that medical students generally preferred an integrative approach to conflict resolution, marked by a propensity to compromise and accommodate and a reluctance to engage in competitive behaviors. In addition to the influence of hierarchical structures within medical teams, the predominance of female participants (72%) likely also contributed to these tendencies (34). At the same time, this distribution closely mirrors the actual gender ratio among medical students in Germany (35), suggesting that the findings can be considered representative in terms of gender composition. Comparable tendencies were observed in a recent study of medical students in the United States, in which accommodating was identified as the most common conflict resolution style (36). These tendencies are further emphasized by a comparison to master’s students in the United States: Medical students tended to choose the accommodating approach more frequently (90th percentile on average) when resolving conflicts with peers, whereas they opted for competing less often (30th percentile on average) than the reference group (32).

According to the model of conflict management strategies, a heightened tendency to accommodate carries the risk that underlying substantive issues remain unresolved, potentially compromising the quality of collaborative work (10). In medical teams, this may lead to treatment errors going unaddressed or conflicts being avoided at the expense of patient care quality: Studies in the German-speaking and international contexts have shown that medical students may remain silent despite perceived patient safety concerns, with hierarchical ward structures identified as an important barrier to speaking up. These findings underline the need to critically consider highly accommodating or conflict-avoidant tendencies in clinical training settings (37–39).

In contrast, the competitive approach to conflict resolution is often understood as the assertive pursuit of one’s own interests. Within the context of the medical profession, this does not merely reflect personal ambition but also encompasses the responsibility to advocate for patient safety (25).

### 6.2 Gender differences in stress perception

In our study, female participants experienced significantly higher stress levels (B = –5.652, *β* = −0.140, *p* = 0.045). The finding is consistent with previous research showing gender differences in stress and burden perception among medical students, with female students often reporting higher levels of perceived stress, psychological distress, or study-related demands (40). Possible explanations discussed in the literature include differences in stress perception and reporting, coping strategies, perceived performance pressure, and psychosocial factors such as self-esteem and optimism (40–42). These findings underscore the need for accessible student support services and curricular stress-prevention measures that are sensitive to gender-related differences in perceived burden and coping while remaining available to all students (43, 44).

### 6.3 Association with preferred conflict style

In line with the initial hypothesis, the findings supported the assumption that medical students’ preferred conflict resolution strategies were associated with perceived stress levels. Specifically, a higher tendency toward compromise was associated with lower stress, whereas a propensity for avoidance was more likely to correspond with increased worry. The relevance of avoidant behavior has also been demonstrated in other studies involving medical teams (8, 22). One explanation is the continued persistence of unresolved conflicts (22). Regarding this aspect, a study conducted with medical residents demonstrated that increased avoidant behavior served as a negative predictor for the quality of work performance (28).

One possible explanation for the reduced perception of stress associated with greater willingness to compromise may be found in the theoretical framework, as compromises allow for the partial satisfaction of both others’ and one’s own needs without the need for in-depth problem analysis (25). This can result in an efficient use of time and resources. Particularly for medical teams, suboptimal working conditions with insufficient resources have been identified as a significant factor contributing to increased conflict (3). Additionally, it has been established that a greater willingness to compromise is more commonly demonstrated by individuals with higher emotional intelligence (29). In conclusion, fostering a greater willingness to compromise in the future could be considered a potentially relevant educational target to better prepare students for real-world working conditions.

### 6.4 Association with prior exposure to team conflicts

Finally, the findings were consistent with the assumption that team conflicts are associated with higher perceived stress for advanced medical students and are related to higher overall stress levels among participants. One possible explanation for this is that, due to common hierarchical disparities, students may not feel confident in asserting their own experiences (45). In qualitative analyses of conflict causes, medical students also identified hierarchy and power imbalances as the most common reasons (2).

### 6.5 Limitations and directions for future research

The limitations of the study stem from the subject matter and the chosen survey design. The management and resolution of conflicts involve complex interpersonal processes that can only be approximated by theoretical models. Individuals’ conflict behavior is fundamentally influenced by multiple factors such as personality structure, individual competencies, role within the team, and the extent of support and communication within the team (3, 8, 27, 29).

Nevertheless, enduring tendencies can be detected through assessments (22, 46), and various studies have demonstrated the effects of conflict resolution strategies on the quality of team performance (3, 22).

A digital cross-sectional survey can only offer limited insight into the cognitive processes of respondents, capturing their attitudes and perceptions at a single point in time. Due to the inherently anonymous nature of participation, the accuracy of self-reported demographic data cannot be guaranteed. Although previous research has shown that the measurement of conflict management tendencies is generally robust against systematic response biases (31, 32), it should be noted that students anticipated discussing their own tendencies in the seminar following the questionnaire. As an additional measure, we, therefore, offered voluntary participation in the discussion. The relatively low explanatory power of the regression model indicates that conflict resolution styles and prior team conflict explain only a limited portion of perceived stress. This is consistent with previous research showing that stress and well-being among medical students are influenced by multiple individual and contextual factors, including coping resources, personality traits, social support, workload, and the clinical learning environment (47, 48).

The interpretation of the regression analysis, particularly the regression coefficients, is limited by the use of Likert scale composite scores. Given that these scales are only quasi-metric, absolute predictions or precise quantitative statements (e.g., percentage values) are not appropriate. Rather, the results should be understood as indicative trends related to the individual predictor variables—an aspect that was carefully considered throughout the analysis by avoiding comparisons of the absolute effects of the different factors.

Additionally, the robustness of the observed effects is strengthened by the substantial sample size (*n* = 219) and the application of standardized, validated German-language assessment instruments.

### 6.6 Future research questions

It remains an open question to what extent individual conflict resolution tendencies and other influencing factors of interpersonal conflict behavior can be modified through educational interventions. Pilot studies have indicated that students’ self-confidence increased following targeted conflict management training (12), and participants reported feeling capable of applying the strategies learned (49). However, Almost et al. (3) have highlighted, in their review, the insufficient exploration of interventions and their potential to mitigate conflict-related burdens by addressing influencing factors.

## 7 Conclusion

In summary, in this study, both the prevalence of team conflicts and individual tendencies in conflict solution were associated with persistent stress experienced by medical students throughout their studies.

There is a significant need for educational interventions in the area of conflict management (1, 49). A key challenge lies in addressing the specific aspects of conflict management within medical teams, where often stressful working conditions complicate effective conflict resolution. As demonstrated in this study, future considerations in medicine should include strengthening medical leadership roles that, amid an increasingly female workforce and constrained time and personnel resources, encourage individual tendencies toward integrative conflict resolution that have the potential to reduce the overall frequency of conflicts (3). At the same time, training programs should address the potentially problematic impact of excessive accommodation on patient safety. Educational interventions for medical students offer a valuable opportunity in this regard, as they often observe medical errors without voicing their concerns (50). The authors therefore emphasize the need to focus on team conflicts as a central source of stress in medical practice and to use self-reflection as an essential training tool.

## Data Availability

The raw data supporting the conclusions of this article will be made available by the authors, without undue reservation.
